# Cognitive Function among World Trade Center-Exposed Community Members with Mental Health Symptoms

**DOI:** 10.3390/ijerph19063440

**Published:** 2022-03-15

**Authors:** Rebecca Rosen, Yongzhao Shao, Qiao Zhang, Jia Bao, Yian Zhang, Arjun Masurkar, Thomas Wisniewski, Nina Urban, Joan Reibman

**Affiliations:** 1 Department of Psychiatry, NYU Grossman School of Medicine, New York, NY 10016, USA; urbann@nychhc.org; 2 World Trade Center Environmental Health Center, NYC Health+Hospitals, New York, NY 10016, USA; qiao.zhang@nyulangone.org (Q.Z.); jia.bao@nyulangone.org (J.B.); yian.zhang@nyulangone.org (Y.Z.); joan.reibman@nyulangone.org (J.R.); 3 Department of Environmental Medicine, NYU Grossman School of Medicine, New York, NY 10016, USA; 4 Department of Population Health, NYU Grossman School of Medicine, New York, NY 10016, USA; 5 NYU Alzheimer’s Disease Research Center, New York, NY 10016, USA; arjun.masurkar@nyulangone.org (A.M.); thomas.wisniewski@nyulangone.org (T.W.); 6 Department of Neurology, NYU Grossman School of Medicine, New York, NY 10016, USA; 7 Department of Medicine, NYU Grossman School of Medicine, New York, NY 10016, USA

**Keywords:** World Trade Center, cognitive impairment, trauma, PTSD, toxin exposure, MoCA, cognition, MCI

## Abstract

The World Trade Center Environmental Health Center (WTC EHC), is a federally designated clinical center of excellence for surveillance and treatment of WTC disaster exposed community members (WTC Survivors). Cognitive impairment (CI) has been extensively described in WTC responders and a concern for progressive impairment in all WTC disaster exposed groups has been raised. Cognitive status, however, has not been systematically characterized in the WTC Survivor population. We describe cognitive status in a subgroup of the Survivor population referred for mental health evaluation (N = 480) in the WTC EHC as measured by scores on the Montreal Cognitive Assessment (MoCA) instrument, and examine their association with WTC exposures and individual-level covariates including PTSD and depression screening inventory scores. In regression analyses, probable cognitive impairment (MoCA score < 26) was found in 59% of the study subjects and was significantly associated with age, race/ethnicity, education, income, depression and PTSD scores. Being caught in the dust cloud on 11 September 2011 was significantly associated with cognitive impairment even after controlling for the above. These data suggest an association with cognitive dysfunction in WTC Survivors with exposure to the toxic dust/fumes and psychological stress from the 9/11 terrorist attack and warrant further systematic study.

## 1. Introduction

Cognitive impairment (CI) is a significant health concern among individuals exposed to dust/fumes and psychological stress from the World Trade Center (WTC) disaster. Extensive studies of those involved in the rescue and recovery efforts after 11 September 2011 (WTC Responders) indicate that WTC exposures may be associated with neuroinflammation, neurodegeneration and increased cognitive impairment, and in turn, with increased risk of Alzheimer’s disease and related dementia in subsequent years [1,2,3,4,5,6,7,8]. In particular, cognitive impairment among WTC Responders has been extensively studied using validated instruments for measuring objective cognitive dysfunctions including the Montreal Cognitive Assessment (MoCA, [9]), and in-depth assessments using well-established blood-based biomarkers and neuroimaging markers of cognitive impairment [1,2,3,4,5,6,7,8]. These reports also include a large number of association studies of cognitive impairment with WTC exposures and associated mental health conditions among WTC Responders. In contrast, fewer published studies report on cognitive status of community members exposed to the WTC dust and fumes. Studies of community members in the WTC Health Registry report self-reported confusion or memory loss associated with cognitive reserve and mental health conditions [10,11]. However, these studies are largely limited to self-reported confusion and memory loss, reinforcing the need for corroborative objective measures of cognitive impairment with validated and widely used instruments such as the MoCA, and in-depth biomarker studies. To date, the impact of WTC exposures on assessment-measured, objective cognitive function in community members (WTC Survivors) exposed to the WTC dust and fumes, and traumatic experiences, has not yet been systematically described.

The terrorist attack on the WTC on 11 September 2001 created an environmental disaster with the collapse of the WTC towers. Local community members had potential for substantial acute dust inhalation from the massive clouds created as the WTC buildings collapsed (WTC dust cloud), as well as chronic inhalation and topical exposure from re-suspended dust and fumes from the fires that burned for months [12,13]. Exposed community members include local residents, local workers (including those who escaped the towers), students, tourists, and commuters passing by on 11 September 2011. Many witnessed death and destruction and feared their own death as they escaped collapsing buildings or were engulfed by blinding dust clouds. Many were displaced from homes and workplaces and/or witnessed prolonged rescue and recovery, with a chronic negative emotional impact. These community members are called WTC Survivors under the H.R.847—James Zadroga 9/11 Health and Compensation Act of 2010 (Zadroga Act). Well-described adverse medical and mental health effects in this population include aerodigestive symptoms [14,15,16,17,18], cancers [19,20,21], neurologic disorders [22,23], cardiovascular disease [24,25] and mental health symptoms such as those that comprise posttraumatic stress disorder (PTSD) and depression [15,26,27,28,29], as well as alcohol use disorders [30,31].

A significant body of research has explored associations between psychopathology and cognitive impairment. Studies have consistently shown depression [32,33,34] and anxiety [35,36] to be associated with cognitive impairment and dementia. Studies have also found PTSD to be associated with cognitive impairment in both military and non-military populations [4,5,7,10,37,38,39,40,41,42]. Extensive research has been carried out on cognitive functioning and decline in the WTC Responder population including on self-reported cognitive complaints [10,41], an association with PTSD [4,5,7], dust exposure [1], as well as biomarkers and neuroimaging studies [2,3,8].

The World Trade Center Environmental Health Center (WTC EHC), was established as a clinical center for treatment and surveillance of community members. It was included as a center of excellence for survivors, under the Zadroga Act, as part of the federal WTC Health Program (WTCHP), to provide treatment and surveillance for defined health conditions that resulted from exposure to the destruction of the WTC towers [43]. Twenty years after the WTC terrorist attack, those exposed to the 9/11 disaster are aging. The majority of patients in the WTC EHC are over 60 years old [20] and cognitive decline is becoming an increasing health concern. Understanding the rates, risks, mechanisms, and modifiable health conditions is, therefore, of increasing importance. Whereas extensive studies of cognitive impairment of WTC Responders have revealed multiple WTC exposure-related risk factors including PTSD [4,7,41], there is a lack of in-depth, systematic studies about the cognitive status of WTC Survivors, who include many women and have a different demographic profile and different exposure profiles than the WTC Responders. We now report on findings of the cognitive status in a subgroup of WTC Survivors undergoing mental health evaluation in the WTC EHC and identify factors, including WTC exposures and WTC-related mental health conditions associated with probable CI.

## 2. Methods

To investigate cognitive status, we summarized the rate of probable CI using proportions. To assess association of categorical covariates (e.g., exposure status to WTC dust cloud) and continuous variables (e.g., PCL scores) with probable CI, we first conduct a univariate analysis using a chi-square test for categorical variables and the Mann-Whitney test for continuous variables to assess association of each of individual covariate of interest with status of probable CI, including association of WTC exposures with probable cognitive impairment. Using the variables significantly associated with probable CI (univariate *p* < 0.05), we then conduct multivariable logistic regression analysis to study the association between WTC exposures and the status of probable CI with adjustment of covariates. More technical details can be found in Section 2.3.

### 2.1. Subjects

This study was conducted at the Bellevue Hospital WTC EHC. Enrollment in the WTC EHC requires exposure to acute or chronic WTC dust and fumes, as well as the presence of physical and/or mental health symptoms with diagnoses related to WTC exposures. Both exposures and associated health conditions are defined under the Zadroga Act (https://www.cdc.gov/wtc/eligiblegroups.html, accessed on 17 February 2022). The degree and duration of exposure varies among patients. Patients enrolled in this program undergo a standardized assessment, including a physical and mental health evaluation with standardized screening tools [18]. Patients with elevated inventory scores of PTSD, depression or anxiety identified during a monitoring visit who were willing to undergo a further evaluation were referred for a diagnostic mental health evaluation. Patients can also self-refer for an evaluation to assess for the presence of a WTC-associated mental health disorder [15,26,27,28]. Patients met inclusion criteria and were included in the dataset of this study if they had undergone an initial standardized exposure, medical, and mental health evaluation between August 2012 and December 2018 and the evaluation included administration of the MoCA; [9]). The Institutional Review Board of New York University School of Medicine approved the research database (NCT00404898) and only data from patients who signed informed consent were used for data analysis.

### 2.2. Assessments

During the first clinic appointment, patients completed a multi-dimensional, interviewer-administered questionnaire (IVQ) that included demographic information and characterizations of WTC-related exposure and occupational status [18]. Individuals were classified as positive for dust cloud exposure if they reported having been in the WTC dust created by the collapsing buildings on 11 September 2011. Potential for WTC acute and chronic exposures was also characterized by classification into four additional categories: local resident, local worker, clean-up worker, and other. Other demographic categories queried included date of birth and age on 11 September 2011, race/ethnicity, sex, education level, household income, occupation, and smoking status. Body mass index (BMI) was calculated using information gathered during the initial medical visit.

Diagnostic evaluations included an unstructured clinical interview to assess psychiatric and psychosocial history and current symptoms, as well as the administration of standardized instruments. Diagnostic evaluations were administered by a trained psychiatrist or psychologist to clarify WTC-relatedness of mental health symptoms.

Symptoms of PTSD were assessed with the Posttraumatic Check List-17-S, trauma specific version (PCL; [44,45]). A score ≥44 was considered positive for probable PTSD, and a score <44 was considered negative for probable PTSD [46]. Symptoms of depression were assessed using the Patient Health Questionnaire-9 (PHQ-9; [47]) by scores indicating minimal (0–4), mild (5–9), moderate (10–14), moderately severe (15–19), and severe (20–27) depression. PHQ-9 and PCL scores were analyzed as categorical and continuous scores.

Cognitive function was assessed with the MoCA [9]. The MoCA is a brief screening instrument with high specificity and sensitivity, which assesses domains of attention, concentration, executive functions, memory, language, visuo-spatial skills, abstraction, and orientation. A cut off score <26 (with one point added for education ≤ 12 years) has been shown to have excellent sensitivity with the ability to detect 90% of subjects with mild cognitive impairment (MCI), 100% of subjects with Alzheimer’s disease, with a 87% specificity [9]. The MoCA has been validated in numerous languages, including Spanish [48]. In the rest of this paper, we call subjects with a MoCA score <26 as having probable CI.

### 2.3. Statistical Methods

Categorical variables were summarized using counts and percentages and their group differences were assessed by Chi-square test. Fisher’s exact test was used in place of Chi-square test if an expected cell count was less than 5. Continuous variables were summarized using median and Q1 and Q3. Between-group differences for continuous variables were assessed using Wilcoxon rank-sum test. Multivariable logistic regression was used to quantify the association between patients with normal cognitive and probable cognitive impairment for covariates of interest including PHQ-9 and PCL scores. Multivariable models were constructed using selected covariates that were significant in univariate analyses. The significance level of two-sided test was set to 0.05. All statistical analyses were conducted using SAS, version 9.4 (SAS Institute, Cary, NC, USA). The proportion of missing data is quite low and negligible for all of the variables, so we reported the analysis results based on subjects without missing data for each of the specific analyses in the tables.

## 3. Results

### 3.1. Patient Characteristics

There were 480 patients who met full inclusion criteria and were included in this analysis (Table 1).

The MoCA was administered in English to the majority of the population, and in Spanish to 17% of the group. The median age of the population was 56, with a slightly higher number of females (54%) than males (46%). Non-Hispanic White patients comprised the largest race/ethnicity group (40%), while many were Hispanic (36%) with fewer non-Hispanic Black patients (19%). Over half (52%) had an annual individual income ≤ $30k a year at their initial visit. The majority of the group (62%) reported having been caught in the WTC dust cloud, and local workers comprised the largest exposure category (66%). Most patients were evaluated since they scored positive on a screening inventory or were suspected of having WTC-related PTSD or depression, and indeed, 75% scored positive for probable PTSD (N = 347), with a median score of 54 for the group. The median PHQ-9 score was 14. Severe depression was reported in 18%, moderate or moderate severe depression were each reported in 26%*,* mild depression in 22%, and 7.4% reported no depression.

### 3.2. Characteristics Associated with MoCA Scores < 26

**Figure 1:**
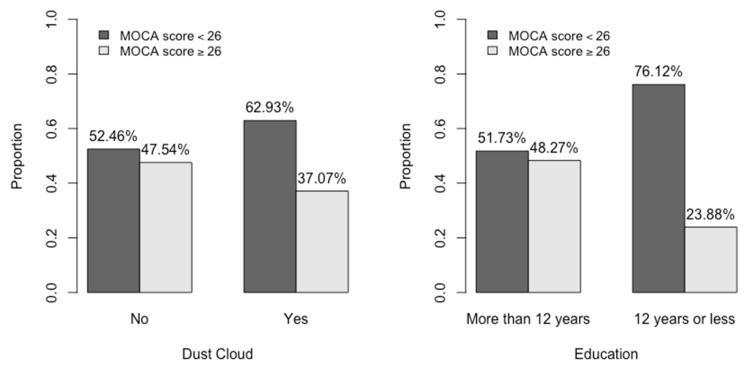
Dust cloud and education level in those with MoCA scores <26 and ≥26.

Over half of the group (59%, N = 281) had MoCA scores < 26, consistent with probable CI and 199 patients had MoCA scores ≥ 26 (Table 1). Using a univariate analysis, we compared demographic characteristics, BMI and smoking status, exposures, and mental health symptom scores of patients with MoCA scores <26 and ≥26. Patients who were older (*p* = 0.02), self-identified as Hispanic or non-Hispanic Black (*p* < 0.001), and whose language was Spanish (*p* < 0.001), were more likely to score in the possible CI category. Patients who had ≤12th grade education (Table 1, Figure 1), as well as those with an income ≤30 K were also more likely to be in the possible CI group (both, *p* < 0.001).

Those who were caught in the dust cloud (*p* = 0.03; Table 1, Figure 1) or worked as a local worker or clean-up worker, were also more likely to be in the probable CI group.

We performed a simple linear regression to predict the continuous dependent variable of MoCA score using PCL and PHQ-9 scores (Table 2).

**Figure 2:**
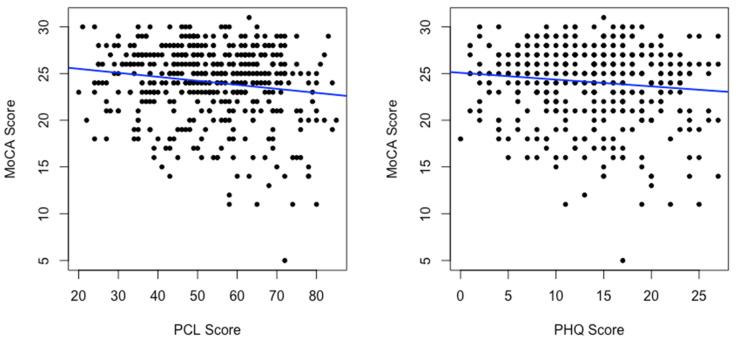
Linear regression of MoCA scores on PCL scores and PHQ scores.

There was a significant inverse association between both MoCA and PCL scores (*p* = 0.001) and MoCA and PHQ-9 scores (*p* = 0.02; Figure 2) consistent with a well-known association between PTSD and CI as well as depression and CI in general populations.

### 3.3. Multivariable Logistic Regression

We performed a multivariable logistic regression analysis to predict status of probable CI with significant predictive variables from the univariate analyses in the model (Table 3).

In the multivariable analysis, age (OR = 1.04; *p* < 0.001), education (OR = 2.01; *p* = 0.02), income, (OR = 1.76; *p* = 0.01), ethnicity self-identified as Hispanic (OR = 1.80; *p* = 0.05) or non-Hispanic Black (OR = 3.01; *p* < 0.001) remained significant. To capture the potential effect of both chronic exposure and/or acute exposure to WTC dust and fumes, we included both WTC exposure categories and status of the acute exposure to the WTC dust cloud on 11 September 2011 in our analysis. The WTC exposure categories was a significant predictor of low MoCA scores univariately, but was no longer statistically significant after adjusting for race, language, education, income and other covariates in the multivariable model. Importantly, those who were caught in the dust cloud on 11 September 2011 were also more likely to be in the probable CI group (OR = 1.59; *p* = 0.04) even with adjustment of other covariates.

## 4. Discussion

This study of the cognitive status of Survivors at the WTC EHC focused on those who underwent diagnostic evaluation for mental health symptoms. We report a high rate of probable CI as measured by the MoCA in the study cohort overall. Furthermore, we report an association of probable CI with demographic characteristics, PTSD and depression symptoms, and acute WTC dust exposures.

Our findings are consistent with existing studies of WTC Responders and support the hypothesis that the WTC dust toxins and traumatic experience, and potentially the resulting PTSD and other medical and mental health comorbidities, have had an adverse impact on cognitive function [1,4,6,7,41]. However, the WTC Responder population may have different rates and risks for CI given that they are predominantly male, white, trained for disaster response, and had different patterns of exposure to the destruction. Our data show support for a detrimental cognitive impact on our untrained civilian population which is diverse, nearly 50% women, comprised of many races and ethnicities, and has a wide range of education and socio-economic status. Indeed, in both univariate and multivariable analysis, age, race/ethnicity, low income and low education attainment remained associated with probable CI. This finding is consistent with current research [49,50,51,52,53] in which these factors are associated with the presence of CI and reinforces the need to include these factors in analysis of CI in an exposed race/ethnically diverse population.

Psychiatric disorders such as PTSD, depression, and anxiety have been reported to be associated with CI in varied ways including as risk factors, as co-occurring, or as comorbid challenges [32,36,51,54]. In our univariate analyses, we also show a significant association between MoCA scores and PCL and PHQ-9 scores, consistent with an association between probable CI and mental health symptoms. However, the Survivors under study can have different exposure profiles than the first responders. For example, the community members may have acute, massive exposure to the dust clouds as they were escaping the towers or leaving offices, and/or long-term, chronic exposures to resuspended dust and fumes in homes, workplaces, and streets [12,13,55]. It is perhaps not a surprise that we show a relatively small magnitude of the slope of the MoCA score and the PCL or PHQ-9; after multivariable logistic analysis, the PCL score was no longer a significant predictor of MoCA score. Moreover, the study group reported here was enriched with patients with elevated mental health scores due to the referral process, resulting in reduced variations of PCL and PHQ-9 scores, which may obscure the relationship between the PCL and PHQ-9 with the MoCA score, as well as reduce their impact as significant predictors of low MoCA scores when included in a multivariable analysis with many other covariates.

We examined exposure to the WTC dust cloud on 11 September 2011 as a component of acute and often massive exposure. Acute exposure to the WTC dust on 9/11 was a significant predictor of MoCA score in both the univariate and the multivariable analysis, suggesting that the environmental exposure might influence the reduction in cognitive function. Many studies of WTC Responders also found that the long duration of exposure is associated with cognitive impairment [1,4,6]. In addition to the adverse impact of acute exposures, the chronic exposures among WTC Survivors due to local residence or work, with potential for exposure to resuspended dust and also fumes from the fires (which burned through the end of December 2001), may also contribute significantly to cognitive impairment. In fact, we previously found evidence that WTC chemicals from chronic exposures were more detectible in blood 12 years after 9/11 than chemicals from acute dust cloud exposures among affected children [56]. Further studies and extensive analysis of the impact of chronic exposures remain to be performed in the future.

Limitations of this study reinforce the need for additional, more systematic research. We studied a population exposed to the same traumatic event—while a strength of the study, this may also limit generalizability of the results. An unexposed comparison group of subjects would be useful in future studies. Also, data on traumatic and significant life events experienced by patients pre- and post-911 were not available for the current study and are important variables for inclusion in future studies due to their potential impact on psychological functioning 20 years post 9/11. Although we found association of acute WTC exposures and cognitive impairment in a group of patients at the WTC EHC, our study cohort is not necessarily representative of the larger Survivor population. These participants were seeking help for mental health complaints within a larger subset of patients seeking care from the WTC EHC, raising the potential for selection bias. Studies of WTC Responders found PCL scores significantly mediate the association between WTC exposures and subjective cognitive impairment [41]. We believe some mediation effect might exist also for the Survivors, however, we did not observe a complete mediation of the PCL scores on the effect of the WTC dust cloud exposure. In other words, WTC dust cloud exposure appears to be an independent risk factor for cognitive impairment in our study in the presence of PCL and PHQ-9 scores as potential mediators. There are multiple potential reasons for the discrepancy between our results and the reported findings of Responders in Singh et al., 2020. First, the WTC exposure profiles differ significantly between the Responders and Survivors, which might impact the mediation effect of the PCL scores on the effect of WTC exposures on cognitive impairment. Secondly, the health impact of the WTC exposures can be quite different between the Responders and the Survivors, as at the time of the disaster, the Responders were mostly healthy men at working age, while Survivors included women, children and elderly civilians. We consider measures of objective cognitive impairment using MoCA rather than self-reported subjective cognitive impairment. Finally, the referral process in this study, which was enriched with study subjects with elevated PCL and PHQ-9 scores, potentially reduced the mediation effect of PCL and PHQ-9 scores on the association between WTC dust cloud exposure and the reduced MoCA scores, warranting further studies in WTC Survivors. Other important variables that were not included in the study can also impact cognitive functioning such as comorbid respiratory symptoms that can be caused by diseases such as asthma [57,58]. Chronic persistent lower respiratory symptoms and severe lung function impairment are very common among patients at WTC EHC and a large portion of WTC Survivors, and it is therefore of clinical relevance to further study the relationship between WTC exposures, persistent respiratory symptoms and lung function impairments with potential cognitive decline among WTC Survivors. Other prevalent comorbidities in this population could have a detrimental impact on cognitive function such as neurological symptoms [22,23], cardiovascular disease and diabetes [51]. Genetic and epigenetic factors such as apolipoprotein E (ApoE) genotype are known to modify the risk and rate of cognitive decline and might impact the probable CI via gene-environment interactions [59,60]. In addition, we do not have baseline cognitive function measures in this population, making additional longitudinal cognitive measurements important. Our findings suggest a need for more thorough neuropsychological testing and cognitive assessment as well as biomarkers in the WTC survivors including the vulnerable subpopulation of WTC Survivors with chronic comorbid mental and physical health disorders.

## 5. Conclusions

In this first study of the cognitive status in the WTC Survivor cohort at the WTC EHC, we report a high rate of probable CI in a population seeking mental health assessment. Given that mental health complaints are very common among WTC Survivors, probable CI should be further studied and the rate of probable CI among WTC Survivors warrants unbiased assessment in better-designed, future studies, including those with comparison groups without WTC exposures. As reviewed by Clouston and colleagues [1], there are multiple plausible pathways underlying the mechanisms by which WTC exposures can impact mental and physical conditions and cognitive impairments. Our results suggest an association of probable CI with demographic characteristics. Importantly, we identified an association of probable CI with acute exposure on 9/11 to WTC dust cloud exposure, which has been reported to contain neurotoxins and has been previously found associated with neurologic disorders in this cohort [22,23]. This study indicates exposure to the WTC dust cloud on 11 September 2011 is an independent risk factor for probable CI in multivariable models adjusting for covariates and potential mediators including PCL and PHQ-9 scores. In particular, we also found that elevated PCL scores and PHQ-9 scores are associated with reduced MoCA scores, despite the enrichment of PTSD and depression symptoms in the study group. Our findings reinforce the need for longitudinal cognitive evaluations in the overall population to detect those with progressive decline to help facilitate early detection and intervention of modifiable contributors. Gene-environment interactions including the roles of the ApoE genotype and acute and chronic WTC exposure on probable CI remain to be studied. Importantly, to understand the biological mechanism of WTC exposures on cognitive impairment as suggested in literature [1], plasma-based neuropathological markers, more cost-effective than neuroimaging, can be used to investigate neuroinflammation, neurodegeneration and associated cognitive decline with potential to identify targets for therapeutic interventions. This would be feasible for the Survivors as it has been successfully shown in blood-based biomarker studies of CI and neurodegeneration among WTC Responders [8]. The cognitive status of these vulnerable subgroups of WTC Survivors remains an important knowledge gap. Consequently, twenty years after 9/11, further study of cognitive status and other aging related health issues will assist in understanding and treating the unique needs of this population.

## Figures and Tables

**Table 1:** Characteristics of study subjects (N = 480) and univariate analysis of MoCA score.

	Total (N = 480)	MoCA ≥ 26 (N = 199)	MoCA < 26 (N = 281)	*p* Value
**Age**				0.022
Median	56.4	54.6	56.9	
Q1, Q3	48.9, 62.7	46.0, 62.2	50.3, 63.2	
**Gender, *n* (%)**				0.562
Female	259	111 (42.9)	148 (57.1)	
Male	221	88 (39.8)	133 (60.2)	
**Race/Ethnicity, *n* (%)**				<0.001
Asian	20	9 (45.0)	11 (55.0)	
Hispanic	170	54 (31.8)	116 (68.2)	
NH-Black	91	27 (29.7)	64 (70.3)	
NH-White	187	104 (55.6)	83 (44.4)	
Other	5	2 (40.0)	3 (60.0)	
**BMI**				0.161
Median	28.10	27.50	28.50	
Q1, Q3	24.6, 31.9	23.9, 31.4	24.9, 32.4	
**Smoking, *n* (%)**				0.285
Current smoker	53	20 (37.7)	33 (62.3)	
Former smoker	148	69 (46.6)	79 (53.4)	
Never smoker	278	109 (39.2)	169 (60.8)	
**Occupation, *n* (%)**				0.190
Disability	47	15 (31.9)	32 (68.1)	
Not working	99	39 (39.4)	60 (60.6)	
Retired	54	18 (33.3)	36 (66.7)	
Working	276	124 (44.9)	152 (55.1)	
**Language, *n* (%)**				<0.001
English	398	184 (46.2)	214 (53.8)	
Spanish	80	14 (17.5)	66 (82.5)	
**Education, *n* (%)**				<0.001
>12 years	346	167 (48.3)	179 (51.7)	
≤12 years	134	32 (23.9)	102 (76.1)	
**Income, *n* (%)**				<0.001
≤$30,000/year	234	79 (33.8)	155 (66.2)	
>$30,000/year	215	108 (50.2)	107 (49.8)	
**PCL-17, *n* (%)**				0.346
Negative	117	53 (45.3)	64 (54.7)	
Positive	347	138 (39.8)	209 (60.2)	
**PCL-17 score**				0.013
Median	54.0	50.0	55.0	
Q1, Q3	43.0, 64.0	41.0, 62.0	44.0, 64.0	
**PHQ-9, *n* (%)**				0.356
None (0–4)	35	13 (37.1)	22 (62.9)	
Mild (5–9)	104	48 (46.2)	56 (53.9)	
Moderate (10–14)	125	50 (40.0)	75 (60.0)	
Mod. severe (15–19)	123	55 (44.7)	68 (55.3)	
Severe (20–27)	85	28 (33.0)	57 (67.1)	
**PHQ-9 score**				0.160
Median	14.0	13.0	14.0	
Q1, Q3	9.0, 18.0	9.0, 17.0	9.0, 19.0	
**Exposure, *n* (%)**				<0.001
Clean-up worker	39	6 (15.4)	33 (84.6)	
Other	33	15 (45.5)	18 (54.6)	
Resident	89	54 (60.7)	35 (39.3)	
Worker	318	124 (39.0)	194 (61.0)	
**Dust Cloud, *n* (%)**				0.030
No	183	87 (47.6)	96 (52.5)	
Yes	294	109 (37.1)	185 (62.9)	

**Table 2:** Univariate linear regressions of MoCA scores on PCL/PHQ-9 scores.

	Slope	*p*-Value
PCL	−0.043	0.001
PHQ-9	−0.073	0.016

**Table 3:** Multivariable logistic regression on status of MoCA < 26.

	Odds Ratio	2.5%	97.5%	*p*-Value
Age	1.04	1.02	1.06	0.001
Race				
Asian	2.38	0.83	6.88	0.11
Hispanic	1.80	1.01	3.19	0.045
NH-Black	3.09	1.68	5.68	0.000
Other	2.36	0.36	15.29	0.368
NH-White (reference)	1.00			
Language				
Spanish	1.59	0.65	3.87	0.310
Education				
≤12 years	2.01	1.12	3.63	0.020
Income				
≤$30,000/year	1.76	1.12	2.77	0.014
PHQ-9	0.98	0.93	1.04	0.51
PCL	1.02	1.0	1.04	0.133
Exposure categories				
Clean-up worker	3.02	0.78	11.74	0.110
Other	1.40	0.53	3.66	0.497
Worker	1.62	0.93	2.83	0.089
Resident (Reference)	1.00			
Dust Cloud exposure on 11 September 2011				
Yes	1.59	1.01	2.49	0.044

## Data Availability

The datasets in the WTC EHC are not publicly available but de-identified and anonymized information are potentially available upon reasonable request.
